# Supplementary material for: Controlling a Van Hove singularity and Fermi surface topology at a complex oxide heterostructure interface
Source: Nat Commun. 2019 Dec 4;10:5534. doi: 10.1038/s41467-019-13046-z (PMC6892806; doi:10.1038/s41467-019-13046-z)
Supplement: Supplementary file 1 — Supplementary Information [file 41467_2019_13046_MOESM1_ESM.pdf]

Supplementary Information for Controlling a Van Hove singularity and Fermi surface topology at a complex oxide heterostructure interface

Mori *et al.*

Supplementary Note 1: Effect of oxygen vacancies and core level spectroscopy

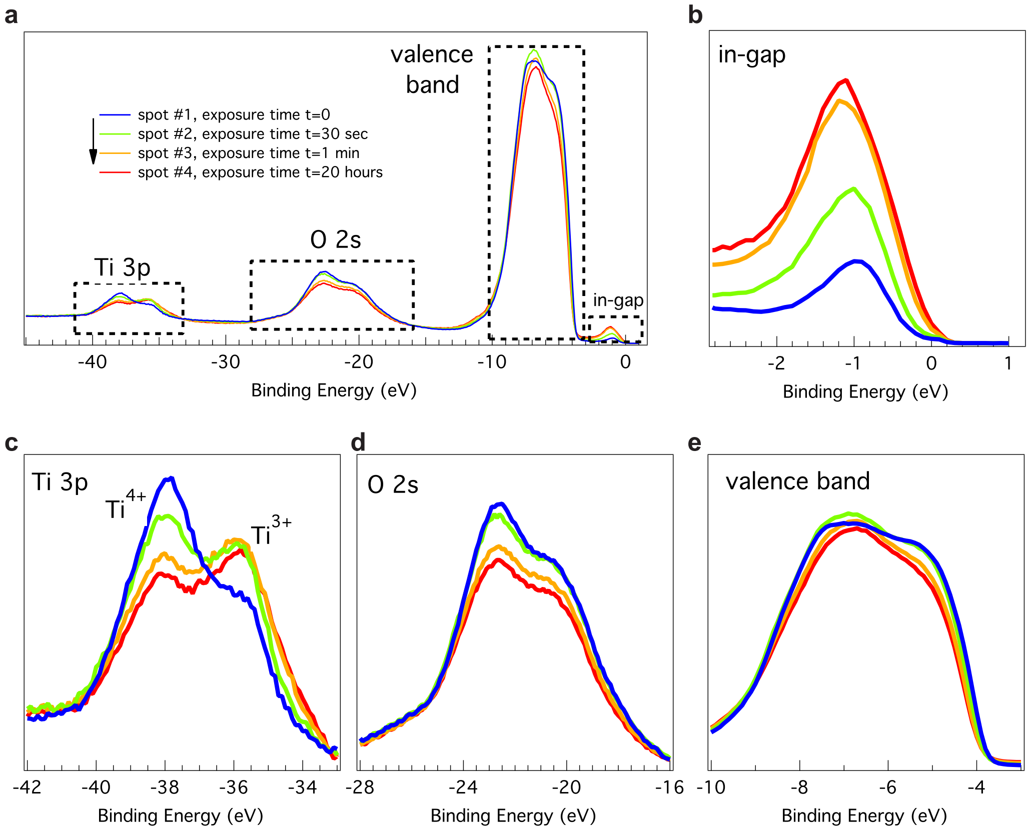

Supplementary Fig. 1. **Core levels and valence bands from photoemission measurements.** **a,** Wide range core-level and valence-level photoemission spectra taken with 92 eV photons, showing four characteristic features: Ti 3*p*, O 2*s*, valence band, and in-gap states. The four dashed-squares frame these four features as indicated. **b-e,** Enlarged narrower range spectra of each characteristic peak, Oxygen vacancy states (in-gap states) (**b**), Ti 3*p* level (**c**), O 2*s* level (**d**), and valence bands (**e**).

Core levels and angle integrated valence bands of the heterostructures of $SrTiO_3$ and $SmTiO_3$ were measured with 92 eV of photon energy (*p*-polarized light). We measured four different spots on the surface of ∞-SrO sample, namely, #1, #2, #3, and #4. Spot #1 corresponds to a non-radiated spot, where the surface had not been exposed to the radiation until the core-level measurements. This spot is considered representative of the "fresh" surface. A short time exposure, $\sim$ 30 sec and $\sim$ 1 min, was done at spot #2 and spot #3, respectively. The spot #4 corresponds to the long-time radiated spot. All the data sets of ∞-SrO shown in the main text were taken at the spot #4 and the core level spectroscopy for this spot was taken after all, giving us an exposure time of about 20 hours. Therefore, we assign the exposure time of synchrotron light t for each spot as follows: $t = 0$ for spot #1, $t \sim 30$ sec for spot #2, $t \sim 1$ min for spot #3, and $t \sim 20$ hours for spot #4. Supplementary Fig. 1a shows the measured spectra of each spot for a wide energy range. There are four distinctive features as marked by four dashed squares, enlarged in Supplementary Fig. 1b–e. Following the exposure time, the intensity of in-gap states,

corresponding to oxygen vacancy states, increased as shown in Supplementary Fig. 1b. The evolution of oxygen vacancy states is confirmed by the peak intensity of O 2s core level decreasing. The core levels of both Ti^{4+} and Ti^{3+} are detected as shown in Supplementary Fig. 1c. While the intensity of Ti^{4+} decreases following the exposure time and the evolution of O vacancy states, Ti^{3+} increases its intensity, suggesting additional carrier doping induced by the radiation of synchrotron light. By comparing these spectra (especially between #2 and #3), it is evident that the change in the core level intensities are saturated quickly (within 1 min of exposure) in our measurements. All data sets in the main text were measured at the saturated condition, thus, we can conclude that the recorded differences of the electronic structures originate from the differences of the sample structures, not from light exposure effects. Also, the observation of Ti^{+4} core level spectra ensures that the we detected photoelectrons coming from the buried $SrTiO_3$ layers.

Supplementary Note 2: Low energy electron diffraction (LEED)

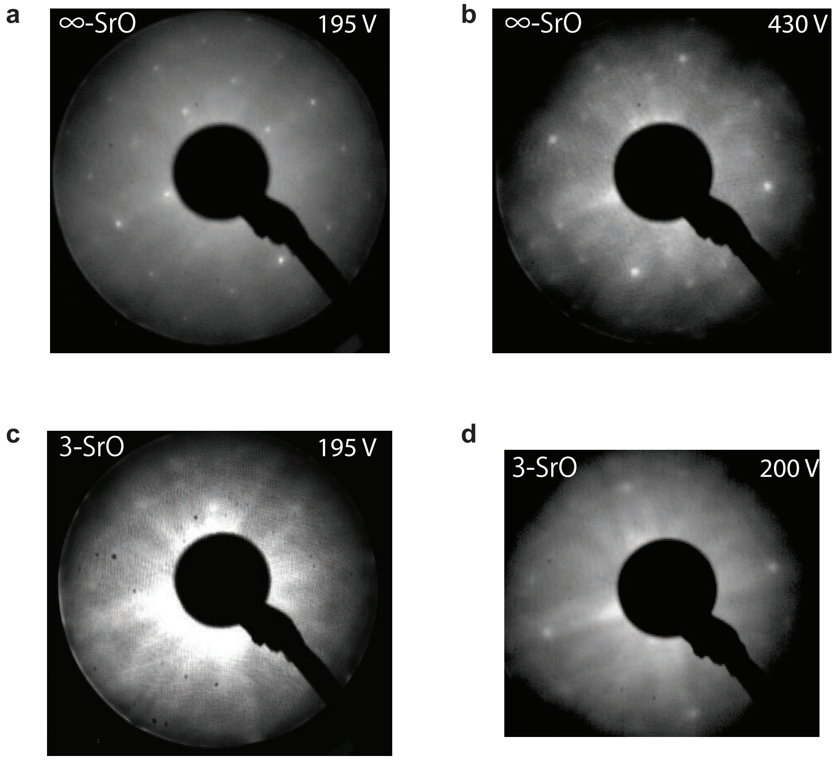

Supplementary Fig. 2. **LEED patterns. a-b,** LEED pattern of ∞-SrO sample taken with an electron voltage of 195 V (**a**) and 430 V (**b**). The data sets for ∞-SrO sample in the main text measured with both s and p polarized light were taken right after this LEED measurement. **c-d,** LEED pattern of 3-SrO sample taken with an electron voltage of 195 V (**c**) and 200 V (**d**). The data sets for the 3-SrO sample in the main text measured with p and s polarized light were taken right after each LEED measurement **c** and **d**, respectively.

LEED measurements were taken after each annealing process to check atomic structures and conditions of near the surface region. Supplementary Fig. 2 shows the various LEED patterns from the different samples, taken before ARPES measurements shown in the main

text. The surface condition for the data sets of the 3-SrO sample with p and s polarized light in the main text corresponds to the LEED pattern in Supplementary Fig. 2c and d, respectively.

Supplementary Note 3: Fermi surfaces along k_z direction

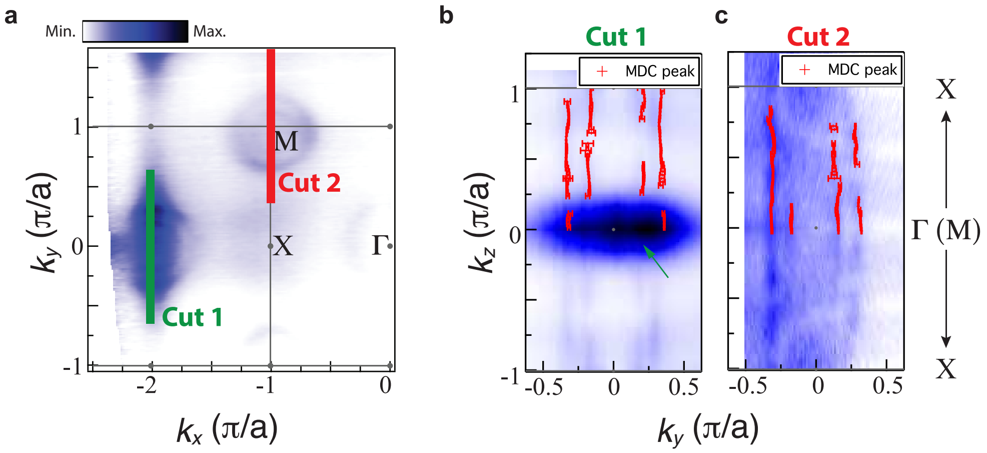

Supplementary Fig. 3. **Fermi surfaces along the out-of-plane (k_z) direction. a,** In-plane Fermi surface maps at E_F for ∞-SrO. The spectra intensity shown here are the sum of the measured intensities with s- and p- polarized light. Cut 1 (green solid line) and Cut 2 (red solid line) represent the location of the out-of-plane cut (k_z-k_y plane) shown in **b** and **c**. **b,c,** Out-of-plane Fermi surface maps E_F for ∞-SrO for Cut 1 (**b**) and Cut 2 (**c**). Cut 1 (Cut 2) represents the k_z dispersion around Γ (M) point in the BZ of $SrTiO_3$. Peak positions and error bars, are obtained by fitting with Lorentzian curves along k_z-direction, are shown with red crosses superposed. For a direct comparison, all Fermi surfaces shown here are centered at $(k_x, k_y, k_z) = (0, 0, 0)$, and shown in a range corresponding to the BZ of $SrTiO_3$. The error bars in **b,c** represent the uncertainties of peak positions from Lorentzian fits.

Supplementary Fig. 3a shows the Fermi surface map of ∞-SrO. To investigate the electronic structures along out-of-plane direction (k_z direction), we measured two distinct cuts (Cut 1 and Cut 2 in Supplementary Fig. 3a) with the various photon energies (78–128 eV). As shown in Supplementary Fig. 3b,c, the d_{xy} states (circle Fermi surfaces at Γ and M points in Supplementary Fig. 3a) has no dispersion along k_z direction, giving an evidence of their two dimensional natures. On the contrary, the $d_{xz/yz}$ states (ellipse Fermi surfaces in the main text), indicated by the green arrow in Supplementary Fig. 3b, has the dispersion along the out-of-plane direction, showing an indicator of the $d_{xz/yz}$ electronic states wave function penetrating slightly into the $SrTiO_3$[1–6]. These observations all resemble and are consistent with the features observed in the two dimensional electron gas of $SrTiO_3$[1, 3, 7] and similar to the two dimensional electron gas of $BaTiO_3$ where the electronic states consist of Ti t_{2g} states.

Supplementary Note 4: Second derivative and EDC of electronic structures

Supplementary Fig. 4a–h represent the second derivatives of our ARPES spectra (see Fig. 2a–d and i–l in the main text). The extracted EDCs at Γ(M) point in BZ for each

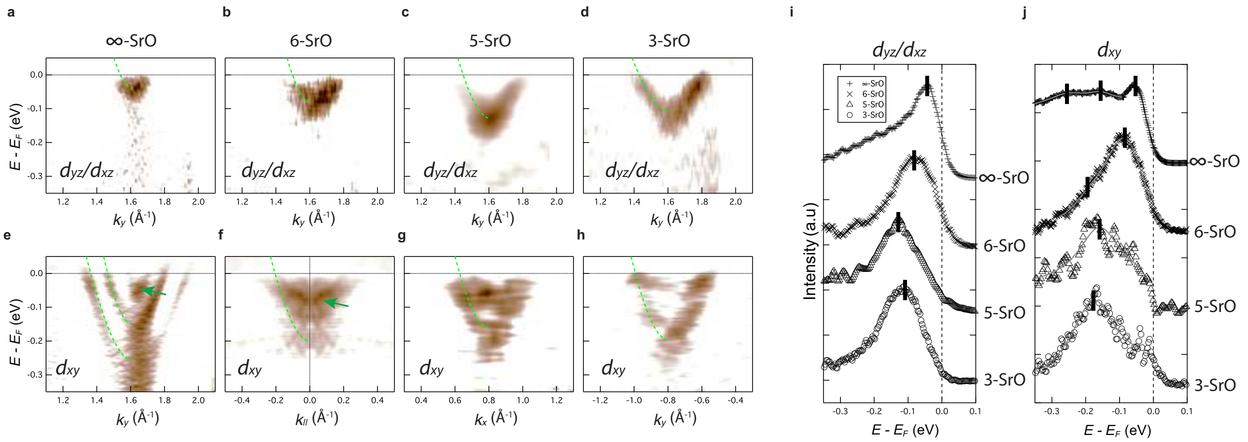

Supplementary Fig. 4. **Second Derivative of spectra and EDC. a–d,** Second derivatives of the ARPES intensities for the light $d_{xz/yz}$ states ($t_{\rm QW} = \infty$- (**a**), 6- (**b**), 5- (**c**), and 3- (**d**) SrO). **e–h,** Second derivatives of the ARPES intensities for the light d_{xy} states ($t_{\rm QW} = \infty$- (**e**), 6- (**f**), 5- (**g**), and 3- (**h**) SrO). The green dashed lines in **a–h,** represent the renormalized band the renormalized experimental band dispersions. **i,j,** EDCs taken at the center of each electronic structure shown in **a–h,**, representing the bandwidth of each structure.

orbital are shown in Supplementary Fig. 4i,j. Note that the peaks closest to E_F in Supplementary Fig. 4j for $t_{\rm QW} = \infty$-SrO and 6-SrO are the $d_{yz/xz}$ states, corresponding to the states marked by the green arrows in Supplementary Fig. 4e,f.

Supplementary Note 5: Interface potential profile

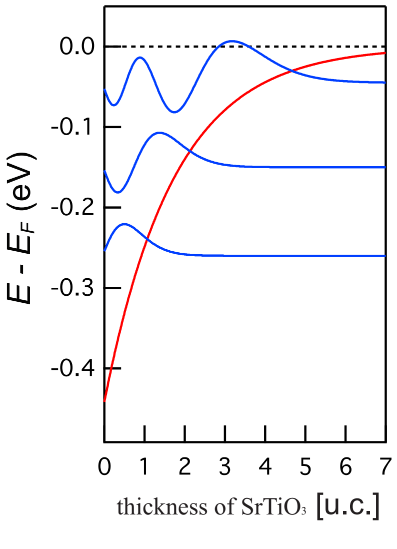

Supplementary Fig. 5. **Estimated interface potential profile.** Interface potential profile estimated by the root of the Bessel function, following the method in Ref[6]

To help understand the interface quantum well, we estimate the potential profile using the

experimental band width as shown in Supplementary Fig. 5. The detail of the calculation is found in Ref[6]. The width of the quantum well in $SrTiO_3$ is estimated to $\sim$13 Å, which is larger than the thickness of 3-SrO ($\sim$11.7 Å), but smaller than the thickness of 4-SrO ($\sim$15.6 Å). Therefore, when the distance of two interface is larger than 6-SrO, the electron liquids in each interface are well separated each other, creating the similar condition to the ∞-SrO structure.

[1] Santander-Syro, A. F. *et al.* Two-dimensional electron gas with universal subbands at the surface of $SrTiO_3$. *Nature* **469**, 189–193 (2011).

[2] Wang, Z. *et al.* Tailoring the nature and strength of electron-phonon interactions in the $SrTiO_3$(001) 2D electron liquid. *Nat. Mater.* **15**, 835–839 (2016).

[3] King, P. D. C. *et al.* Quasiparticle dynamics and spin-orbital texture of the $SrTiO_3$ two-dimensional electron gas. *Nat. Commun.* **5**, 3414 (2014).

[4] Chang, Y. J. *et al.* Layer-by-layer evolution of a two-dimensional electron gas near an oxide interface. *Phys. Rev. Lett.* **111**, 126401 (2013).

[5] Plumb, N. C. *et al.* Mixed dimensionality of confined conducting electrons in the surface region $SrTiO_3$. *Phys. Rev. Lett.* **113**, 086801 (2014).

[6] Moser, S. *et al.* How to extract the surface potential profile from the ARPES signature of a 2DEG. *Journal of Electron Spectroscopy and Related Phenomena* **225**, 16–22 (2018).

[7] Meevasana, W. *et al.* Creation and control of a two-dimensional electron liquid at the bare $SrTiO_3$ surface. *Nat. Mater.* **10**, 114–118 (2011).
